# Editorial: Cellular and Molecular Mechanisms at the Proliferation Stage in Wound Healing: From Scarring to Tissue Regeneration

**DOI:** 10.3389/fcell.2021.659089

**Published:** 2021-02-19

**Authors:** Shiro Jimi, Arman Saparov, Satoshi Takagi

**Affiliations:** ^1^Central Lab for Pathology and Morphology, Faculty of Medicine, Fukuoka University, Fukuoka, Japan; ^2^Department of Medicine, School of Medicine, Nazarbayev University, Nur-Sultan, Kazakhstan; ^3^Department of Plastic, Reconstructive and Aesthetic Surgery, Faculty of Medicine, Fukuoka University, Fukuoka, Japan

**Keywords:** wound healing, regeneration, scar, proliferation stage, cell interaction

Wound healing is a complex physiological reaction in our body to tissue injury that can lead to the impairment of the original organ functions depending on the area of tissue injury. Many cell types participate in the wound healing process, including but not limited to, tissue resident cells, cells of the immune system, vascular cells, fibroblasts, and tissue progenitor/stem cells. However, the cellular and molecular regulatory mechanisms of wound healing are not yet fully identified. In the proliferation stage, granulation tissues develop accompanied by matrix deposition and neovascularization, which lead to proper regenerative responses including epithelialization. If this reaction is impaired, then scar formation and non-regenerative healing may occur, in which case many of aggravating factors, such as growth factors, inflammation, and tensile forces, are involved.

This Research Topic consists of 11 published articles including 5 reviews, 2 mini-reviews, and 4 original research manuscripts. On behalf of the Topic Editors, we thank all the authors for their contribution. With their participation, we could draw a future perspective of wound healing research.

## Cells In Wound Lesions

Faster wound healing is always better for patients, and it is desirable that defected wound lesions heal to regenerate original normal tissue. If fibrous/fibroproliferative tissue develops as hyperplastic scar and keloid, respectively, aggravated appearance and uncomfortableness will affect the patient's quality of life. Although delayed wound healing and fibrous/fibroproliferative healing are contradictory biological reactions, both provide serious issues in wound healing in clinical practice. Limandjaja et al. provide important knowledge regarding abnormal fibrosis, keloid. The review article details current research status and classification. The authors emphasized on several pathophysiological factors for keloids, such as inflammation-related environmental factors, keloid-prone topological factors and genetically predisposed individual factors. Defining the factors that contribute to the formation of wound lesion necessitates a demand for future basic and clinical research.

In the process of keloid development, abnormal cellular functions have been investigated, and fibroblasts are a major cell type that are responsible for fibrosis-prone lesions. Although endothelial cells are also known to be a key player not only in inflammation, but also in fibrosis, their functional analysis in keloids has not been done. Matsumoto et al. utilized a gene expression analysis of human endothelial cells isolated from keloid and normal skin in patients by using magnetic-activated cell sorting and identified that SERPINA3 and LAMC2 were overexpressed in keloid endothelial cells. Overexpression of the indicated genes may affect keloid pathogenesis by inhibiting matrix degradation and prolonging inflammation. However, additional experiments should be performed to confirm this.

Macrophages are multifunctional innate immune cells with scavenging/phagocytosis as their main function. In addition, they play an orchestrating role in the wound healing process. Barman and Koh summarized current knowledge of macrophage function in the pathogenesis of diabetes. Certain types of macrophages with pro-inflammatory and pro-healing phenotypes may play a dominant role during wound healing, whereas dysregulation of macrophage polarization could cause impaired wound healing. Thus, it is important to elucidate the relationship between macrophage polarization and the exacerbating factors in unhealed wounds.

## Wound Healing Therapy

New and effective biomaterials for wound therapy are desirable, which may lead to more effective treatment of unhealed wounds. In this Research Topic, Nurkesh et al. reviewed recent findings on using biomaterials, as drug delivery systems, to extend the activity of incorporated growth factors/cytokines for improving wound healing. Singampalli et al. described the roles of anti-inflammatory cytokine IL-10 and glycosaminoglycan hyaluronan in reducing scarring and improving tissue regeneration. A zwitterionic betaine-incorporated collagen sponge was newly designed for anti-oxidation and anti-inflammation by Chen et al.. Another approach in wound therapy is using a ubiquitin-specific peptidase 15 to enhance re-epithelization via an increase in keratinocytes that was reported by Zhao et al. in addition to the activation of TGF-β signaling pathway in fibroblasts. Development of new biomaterials/molecules may reveal a future strategy for wound healing therapy.

Collagen exists as an extracellular matrix protein and cellular scaffold in granulation tissue. However, its excessive deposition leads to the development of fibrous/fibroproliferative lesions in wound healing. Sato et al. described a novel population of mouse fibroblasts segregated by p75NTR expression and summarized their findings. The authors proposed that collagen degraded di-peptide, Pro-Hyp that arises in wounded tissue or from the blood via food intake, triggered fibroblast transition from a growth arrested subtype to a growth prone p75NTR-positive subtype. This new concept regarding collagen peptides in wound healing should be verified in the future.

The application of MSCs isolated from bone marrow or fat tissues has emerged as another approach for wound therapy. The review article by Jiang and Scharffetter-Kochanek suggested that supplemented MSCs may act as an environment sensing adaptive cells in granulation tissues during wound healing. It was recently reported that microtube-based organelle called primary cilia senses chemical and mechanical signals by Hosio et al. This paracrine effect, rather than their self-renewal and differentiation capacity into several lineages, meet the changing demands during wound healing progression.

## Animal Model

Many types of animal models for wound healing are currently available. However, a scar forming model in rodents is rare. Marchesini et al. created a wound scar model in rats using talc. This animal model is useful for studying the pathogenesis of hypertrophic scar formation and to assessing potential therapy for treatment.

## Conclusion

The collection of papers published in this Research Topic further contributes to our understanding of the wound healing process and describes new approaches to reduce scarring and improve wound healing. We hope these studies will stimulate further research in this challenging area of wound healing.

## Author Contributions

All authors listed have made a substantial, direct and intellectual contribution to the work, and approved it for publication.

## Conflict of Interest

The authors declare that the research was conducted in the absence of any commercial or financial relationships that could be construed as a potential conflict of interest.

